# Laparoscopic Sugarbaker Repair of Parastomal Hernia with Gastric Incarceration: A Case Report and Review of the Literature

**DOI:** 10.70352/scrj.cr.25-0693

**Published:** 2026-01-23

**Authors:** Masatsugu Kojima, Toru Miyake, Soichiro Tani, Keiji Muramoto, Sachiko Kaida, Katsushi Takebayashi, Hiromitsu Maehira, Reiko Otake, Haruki Mori, Nobuhito Nitta, Tomoharu Shimizu, Masaji Tani

**Affiliations:** 1 Department of Surgery, Shiga University of Medical Science, Otsu, Shiga, Japan; 2 Medical Safety Section, Shiga University of Medical Science Hospital, Otsu, Shiga, Japan

**Keywords:** parastomal hernia, stomach incarceration, Sugarbaker technique, stomach obstruction, laparoscopic surgery

## Abstract

**INTRODUCTION:**

Parastomal hernia is a common complication of stoma creation; however, gastric involvement is extremely rare, with only approximately 2 dozen cases reported. Gastric incarceration in a parastomal hernia can cause severe complications, including gastric outlet obstruction and ischemia, and requires timely surgical management.

**CASE PRESENTATION:**

We describe the case of a 57-year-old obese female who underwent transverse colostomy for ischemic colitis and presented with upper abdominal pain and vomiting. She had a history of Buerger’s disease, bilateral lower limb amputation, central adrenal insufficiency, and recurrent colonic stoma prolapse requiring colonic resections. CT revealed gastric outlet obstruction due to stomach incarceration within the parastomal hernia sac. After stabilization of her general condition and nasogastric decompression, she underwent laparoscopic repair 18 days after admission. Intraoperatively, the stomach was incarcerated by traction on the gastrocolic ligament. The gastrocolic ligament was divided, and the stomach was dissected from the mesocolon to maintain a safe distance from the stoma and prevent further traction by the colon. The hernia defect was closed using barbed sutures, followed by laparoscopic Sugarbaker repair with mesh placement. Her postoperative course was uneventful, and no recurrence was observed at 10 months of follow-up.

**CONCLUSIONS:**

We present a rare case of parastomal hernia with gastric incarceration that was successfully managed using laparoscopic Sugarbaker repair. Sufficient gastric mobilization, including division of the gastrocolic ligament and dissection from the mesocolon, is essential to ensure mesh coverage of the hernia defect and minimize recurrence risk.

## INTRODUCTION

Parastomal hernia is one of the most common complications of stoma creation, with an incidence reported to range from 0% to 48%.^1)^ Because a stoma is created by exteriorizing the intestine through a fascial defect, the fascia is not closed, and herniation may occur more frequently. Obesity has been identified as a risk factor for hernia development.^2)^ Although the overall incidence of parastomal hernia is high, only 10%–20% of patients develop symptoms that warrant surgical intervention.^3)^ Surgical repair is recommended for symptomatic hernias, and patients presenting with incarceration represent a strong indication for surgical intervention, often on an emergency basis.^4)^ Hernial contents typically include the small intestine, colon, or greater omentum; however, gastric involvement is very rare.^5)^ Stomach-containing parastomal hernias may lead to severe complications, such as gastric outlet obstruction or ischemia due to incarceration, requiring prompt diagnosis and management. Because gastric parastomal hernias are extremely rare, an optimal surgical approach has not yet been established.^5)^ We report a rare case of parastomal hernia with gastric incarceration, which was considered to be caused by traction through the gastrocolic ligament and successfully managed by laparoscopic Sugarbaker repair after stomach mobilization. Additionally, we reviewed previously reported cases and discussed the clinical features and surgical management of this rare condition.

## CASE PRESENTATION

A 57-year-old female with obesity presented to our emergency department with upper abdominal pain and vomiting. She had experienced multiple episodes of vomiting over the preceding 2 days and was unable to maintain adequate oral intake.

**Fig. 1:**
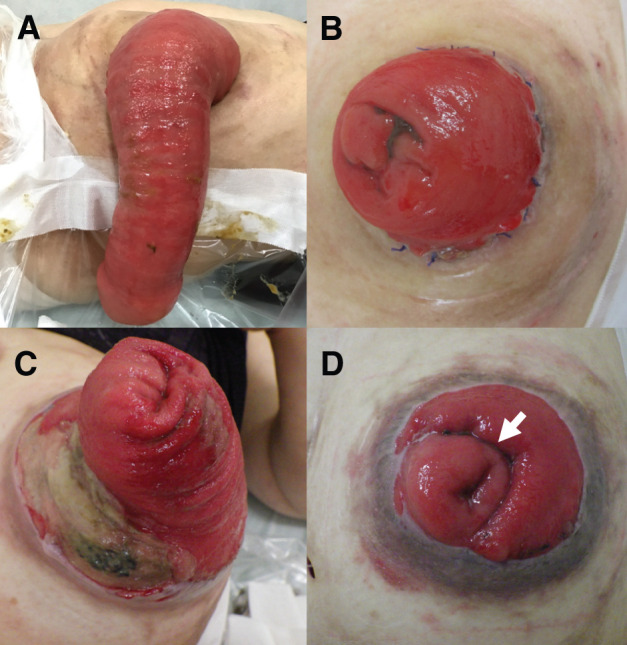
Clinical course of recurrent stoma prolapse. (**A**, **B**) Initial stoma prolapse. The prolapsed bowel segment was resected, and a new stoma was reconstructed using an Altemeier-like technique. (**C**, **D**) Recurrent stoma prolapse occurred, for which bowel resection was performed using a linear stapler to reshape the stoma. The arrow indicates the staple line at the site where the colon was partially resected.

She had a significant medical history of Buerger’s disease, which resulted in bilateral below-knee amputations, central adrenal insufficiency managed with corticosteroids, and multiple abdominal surgeries. Three years earlier, she underwent a left hemicolectomy with transverse colostomy for ischemic colitis. Since then, she experienced recurrent stoma prolapse, which required colonic resections and stoma revisions at 1 year and 3 months prior to presentation (**Fig. 1**).

**Fig. 2:**
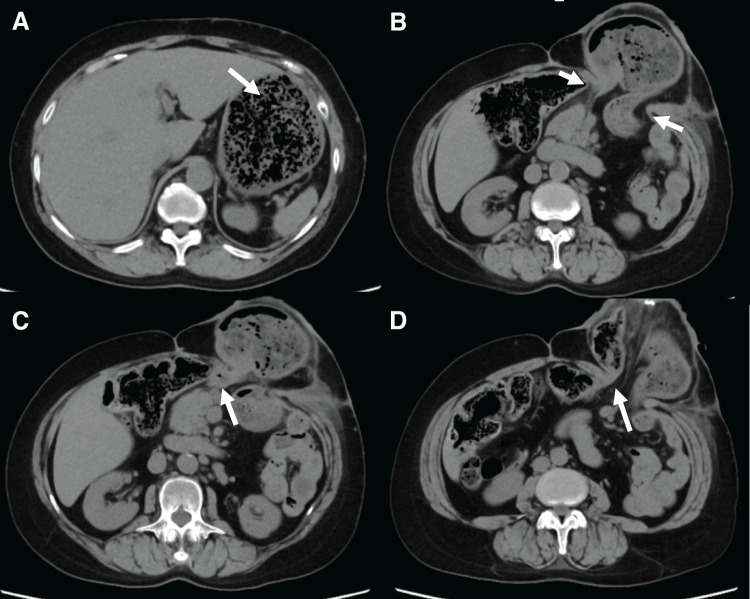
Preoperative CT findings showing gastric incarceration in a parastomal hernia. Axial CT images (**A**–**D**) are shown from cranial to caudal levels. (**A**) Marked gastric dilatation (arrow) was observed. (**B**) The gastric body was incarcerated and herniating through the parastomal defect (arrows). (**C**) Thickening of the gastric wall was evident in the antrum (arrow). (**D**) The lifted colon (arrow) was identified at the caudomedial side of the hernia orifice.

On arrival, the abdomen around the stoma was distended with mild tenderness. She was dehydrated and hypoxemic because of aspiration pneumonia. Laboratory investigations revealed leukocytosis (white blood cell count, 26000/μL), anemia (hemoglobin, 9.6 g/dL), hypoalbuminemia (2.8 g/dL), and acute kidney injury (creatinine, 2.2 mg/dL). CT revealed marked gastric distention secondary to incarceration within the parastomal hernial sac, with evidence of gastric outlet obstruction (**Fig. 2**). The hernial orifice measured 6.5 cm in the craniocaudal direction and 5.5 cm transversely. In addition, pulmonary consolidation consistent with aspiration pneumonia was observed.

After stabilization of the patient’s general condition with intravenous fluids, nasogastric decompression was achieved via gastric tube placement, and she was started on antibiotic therapy and a proton pump inhibitor. The tube initially drained a large amount of fluid, but the output decreased to a small volume of non-bloody, normal gastric fluid immediately. Her abdominal pain and distension improved, and no peritoneal signs were observed. Laboratory data showed no progression of anemia and improvement in inflammatory markers. Based on these clinical and laboratory findings, we judged that the risk of gastric perforation or ischemic necrosis was low. Therefore, after confirming clinical stability, elective parastomal hernia repair was planned.

Given her history of multiple colonic resections for stoma prolapse, further colonic resection and relocation of the stoma to the right side would have risked excessive shortening of the colon, potentially leading to a high-output stoma. In addition, we were concerned about the possibility of recurrent stoma prolapse due to sagging of the remaining colon. Considering these factors, the laparoscopic Sugarbaker technique was deemed the most appropriate option to prevent both recurrent parastomal herniation and stoma prolapse. The patient subsequently underwent laparoscopic Sugarbaker repair 18 days after admission.

**Fig. 3:**
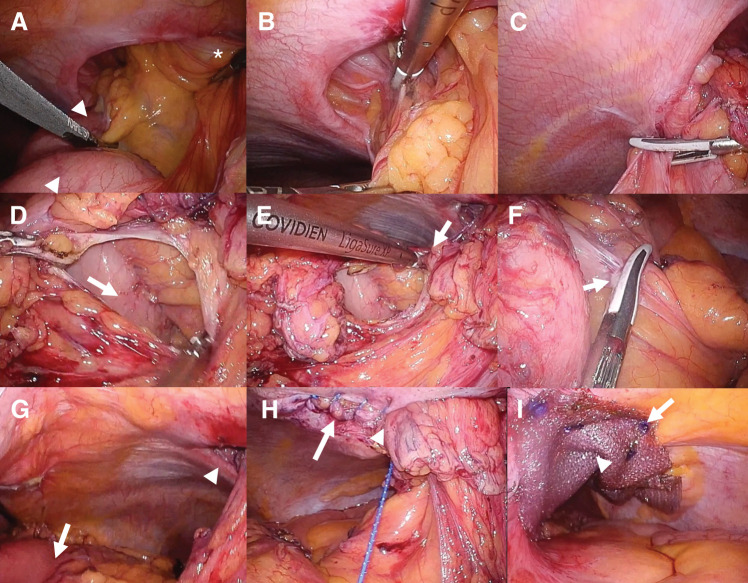
Intraoperative findings of laparoscopic repair for parastomal gastric herniation. (**A**) The herniated stomach (arrowheads) was identified at the cranial side of the colon elevated as stoma (*). (**B**) Dissection was performed between the hernia sac and the stomach. (**C**) The gastrocolic ligament was divided at the level of the hernia orifice. (**D**) An opening was made in the gastrocolic ligament, and the lesser sac was entered, where the dorsal surface of the stomach (arrow) became visible. (**E**) The gastrocolic ligament (arrow) was further divided. (**F**) Physiological adhesions (arrow) between the transverse mesocolon and the stomach were dissected. (**G**) Stomach traction through the gastrocolic ligament was sufficiently released, and the stomach (arrow) was freed from the hernia orifice (arrowhead). (**H**) The hernial orifice was closed using a running barbed suture (arrow) to eliminate potential space while avoiding excessive compression of the mesentery (arrowhead). Closure was performed from the point farthest from the stoma toward the bowel side to allow fine adjustment of tension. (**I**) Sugarbaker repair was performed using an intraperitoneal mesh (arrow) tailored to achieve 5 cm of overlap beyond the suture line and the stoma opening (arrowhead). The mesh also covered the proximal colon.

**Fig. 4:**
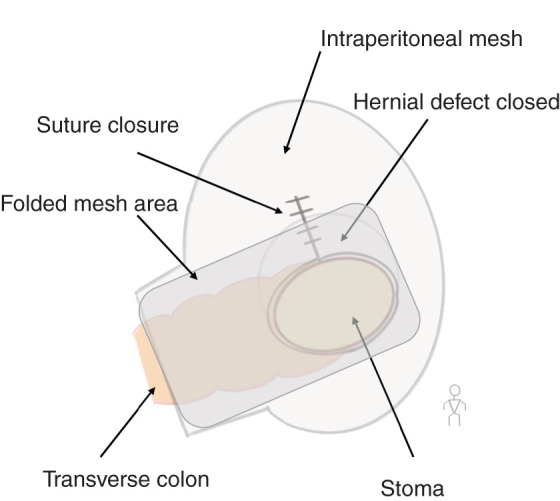
Spatial relationship among the hernial orifice, suture line, and mesh placement. The defect was closed using a continuous barbed suture. A mesh designed for intraperitoneal use was employed, and the portion in contact with the stoma and oral-side colon was folded so that the visceral (intraperitoneal) surface faced inward. The mesh margins were trimmed to ensure a 5-cm overlap from both the stoma and the suture line.

Intraoperatively, the stomach was drawn into the parastomal hernial sac through traction of the gastrocolic ligament (**Fig. 3**). Reduction of the incarcerated stomach was challenging; therefore, the left gastrocolic ligament adjacent to the stoma was divided to facilitate reduction. Subsequently, the gastrocolic ligament was further divided, and the stomach was dissected from the mesocolon to maintain a safe distance from the stoma and prevent further traction by the colon. The hernial defect was located on the cranial and medial side of the stoma. The defect was closed using a running barbed suture to eliminate potential space between the abdominal wall and the elevated bowel and mesentery, while avoiding excessive tension to preserve mesenteric perfusion. The closure was performed from the point farthest from the stoma toward the bowel side to allow fine adjustment of tension. Following defect closure, laparoscopic Sugarbaker repair was performed using an intraperitoneal mesh tailored to achieve 5 cm of overlap beyond the suture line and the stoma opening. The mesh also covered the proximal colon, and the portion in contact with the stoma and proximal colon was folded so that the visceral (intraperitoneal) surface faced inward (**Fig. 4**). Operative time was 191 min with minimal blood loss. Postoperative course was uneventful, and the patient was discharged on POD 12.

**Fig. 5:**
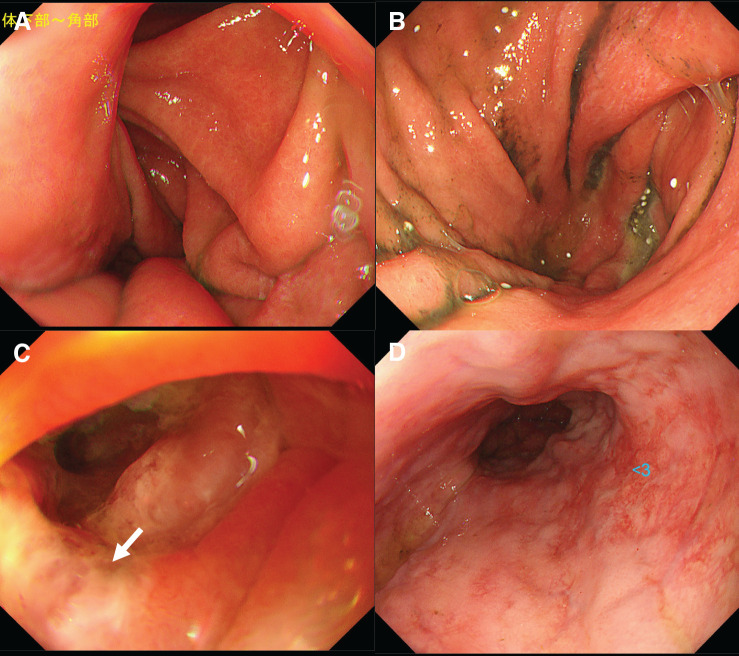
Postoperative upper gastrointestinal endoscopy. (**A**, **B**) Atrophic gastritis of the gastric body and pylorus was observed, with poor distensibility. (**C**) A longitudinal ulcer (arrow) suspicious with ischemic change was observed at the horizontal portion of the duodenum. (**D**) A sliding hiatal hernia with severe inflammation was also identified.

**Fig. 6:**
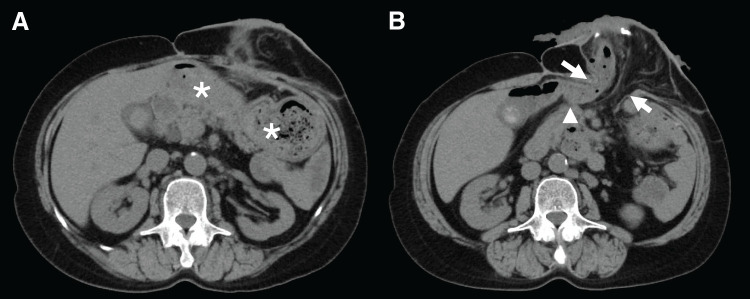
Postoperative CT obtained 2 months after surgery. (**A**) The stomach (*) is located intraperitoneally beneath the abdominal wall and shows no evidence of protrusion or herniation into the parastomal defect. (**B**) Only the elevated colon and its mesentery (arrows) are seen protruding through the abdominal wall as the stoma, with no evidence of parastomal hernia recurrence or stoma prolapse. Although the mesh itself is not clearly visualized, the proximal colon (arrowhead) courses along the abdominal wall.

Endoscopy performed on day 27 revealed chronic gastritis and linear duodenal ulceration, likely ischemic in origin, and severe esophagitis secondary to reflux. The gastric body and antrum also showed impaired distension, which may be related to the effects of incarceration (**Fig. 5**). On the CT obtained 2 months postoperatively, no gastric herniation was observed (**Fig. 6**). Only the elevated colon and its mesentery were seen protruding through the abdominal wall as the stoma, and no recurrence of the parastomal hernia was identified. Although the mesh itself was not clearly visualized, the proximal colon ran along the abdominal wall, which likely represents the appearance of abdominal wall-anchoring achieved by the Sugarbaker technique. No hernia recurrence was observed at 10 months of follow-up.

## DISCUSSION

Parastomal hernia is one of the most common complications of stoma creation, with an incidence reported to range from 0% to 48%.^1)^ Hernial contents typically include the small intestine, colon, or greater omentum; however, gastric involvement is rare.^5)^ A PubMed search using the keywords “parastomal hernia” and (“gastric” OR “stomach”) identified 21 reports that described 22 cases of stomach-containing parastomal hernia, which is summarized in **Table 1**.^5–25)^ The majority of the patients were older adult female, with only one male patient. Their ages ranged between 41 and 93 years, with most patients in their seventies or eighties, accounting for more than half of the reported cases. Twenty patients had end stomas and 2 had loop stomas; among the end stomas, the majority were colostomies, with only 2 ileostomies reported. All hernias occurred on the left side, and no right-sided hernias were identified, likely reflecting the anatomical configuration of the stomach.

**Table 1:** Reported cases of parastomal hernia with gastric incarceration

First author	Year	Age (years)	Sex	Previous surgery	Stoma location	Stoma type	Management	Operative procedure	Operative technique	Outcome
Figiel^6)^	1967	76	F	Colostomy	T/C	Loop	Surgery	Laparotomy	Primary repair	Cancer–related death
McAllister^7)^	1991	91	F	Hartmann	Colon	End	Surgery	Laparotomy	Primary repair, Transposition	No event
Ellingson^8)^	1993	77	F	Hartmann	Colon	End	Surgery	Laparotomy	Primary repair	No event
Bota^9)^	2012	41	F	TPC	Ileum	End	Surgery	Laparotomy	Mesh repair	Mesh infection
Ilyas^10)^	2012	93	F	Hartmann	Colon	End	Surgery	Laparotomy	Primary repair	No event
Ramia-Ángel^11)^	2012	64	F	APR	Colon	End	Conservative	–	–	No event
Marsh^12)^	2013	81	M	Hartmann	Colon	End	Surgery	Laparotomy	Stomach repair, Transposition, Defect enlargement	Wound infection
Barber-Millet^13)^	2014	69	F	Hartmann	Colon	End	Surgery	Laparotomy	Mesh repair, Transposition	No event
Eastment^5)^	2018	91	F	TC	Ileum	End	Conservative	–	–	No event
Bull^14)^	2019	85	F	Colostomy	Colon	Loop	Surgery	Laparotomy	Primary repair, Transposition	No event
Waheed^15)^	2019	58	F	Hartmann	Colon	End	Conservative	–	–	No event
Vierstraete^16)^	2020	74	F	Colostomy	Colon	End	Surgery	Laparotomy	Primary repair, Transposition	Gastroparesis
Vierstraete^16)^	2020	69	F	PE	Colon	End	Surgery	Laparotomy	Mesh repair	No event
Ekowo^17)^	2020	92	F	APR	Colon	End	Conservative	–	–	No event
Anandan^18)^	2020	60	F	APR	Colon	End	Surgery	Robotic	Mesh repair (Sugarbaker)	No event
Centauri^19)^	2020	83	F	Hartmann	Colon	End	Conservative	–	–	No event
Johnson^20)^	2021	68	F	Hartmann	Colon	End	Surgery	Laparoscopic	Stoma closure, Primary repair	No event
Khan^21)^	2022	83	F	Hartmann	S/C	End	Conservative	–	–	No event
Christodoulou^22)^	2022	68	F	Hartmann	Colon	End	Conservative	–	–	No event
Baig^23)^	2022	75	F	Hartmann	S/C	End	Conservative	–	–	Hernia–related death
Bodimeade^24)^	2023	70s	F	Hartmann	Colon	End	Conservative	–	–	No event
González Fernández^25)^	2024	84	F	Hartmann	Colon	End	Conservative	–	–	No event
Our case		57	F	Hartmann	T/C	End	Surgery	Laparoscopic	Mesh repair (Sugarbaker)	No event

Cases labeled “Colon” indicate that the original report did not specify the exact colonic segment.

Transposition indicates relocation and reconstruction of the stoma at a different site.

APR, abdominoperineal resection; PE, pelvic exenteration; S/C, sigmoid colon; T/C, transverse colon; TC, total colectomy; TPC, total proctocolectomy

Surgical treatment was performed in 13 (59.1%) patients, whereas 9 (40.9%) were managed conservatively with nasogastric decompression, which resulted in symptom resolution. Regarding surgical approaches, 10 cases treated by laparotomy were reported up to 2020, whereas since 2020, 3 cases treated with minimally invasive surgery have been reported, including 1 robotic and 2 laparoscopic procedures, one of which was our case. As for operative techniques, primary suture repair was performed in 7 cases, mesh repair in 5, and stoma transposition in 4. Among the mesh repairs, detailed descriptions of the technique were limited; however, both our case and a previously reported robotic case were managed using the Sugarbaker technique.^26)^

Recent evidence suggests that mesh-based repair has lower recurrence rates than primary suture repair, although the risk of mesh-related infections remains a concern.^27)^ Among laparoscopic approaches, the Sugarbaker technique has demonstrated superior long-term outcomes compared with the keyhole method, with significantly lower recurrence rates.^26,27)^ The main advantage of the Sugarbaker method is that both the stoma orifice and proximal bowel can be reinforced by mesh coverage, whereas the keyhole technique only addresses the stoma orifice, leaving a potential gap between the mesh and the bowel.

Although follow-up periods were either not described or appeared short in most reports, hernia-related deaths have been documented in conservatively treated patients. In the operative cases, the reported postoperative complications were limited to one surgical site infection and one mesh infection. Additionally, one patient required gastric repair due to incarceration-related injury, and in our case, inflammatory and ischemic changes were observed extending from the pylorus to the duodenum, presumably associated with gastric incarceration. These observations suggest that gastric incarceration may occasionally result in gastric injury. This possibility, together with the report of one hernia-related death in a conservatively managed patient, should be considered when determining treatment strategies. Although some older adult patients with poor general condition have been managed conservatively without subsequent surgery, a non-operative approach should be adopted with caution, as delayed intervention may potentially increase the risk of gastric ischemia, perforation, and aspiration pneumonia secondary to gastric obstruction. These complications can ultimately lead to deterioration of the general condition.

In the present case, the following factors may have contributed to gastric incarceration. First, repeated stoma prolapse necessitates multiple transverse colonic resections, which allow the stomach to be drawn into the parastomal defect via the gastrocolic ligament, ultimately leading to gastric incarceration. Second, chronic abdominal pressure due to obesity and habitual prone positioning, which had become customary because of lower limb amputation and associated pain extending from the legs to the lower back, likely contributed to the development of the hernia and prolapse. Third, a history of Buerger’s disease with ischemic colitis suggests underlying vascular compromise, which may have predisposed the patient to inflammatory and ischemic changes in the pylorus and duodenum, thereby aggravating gastric outlet obstruction.

There are 2 possible mechanisms underlying the development of stomach-containing parastomal hernias. One is *traction type*, in which the stomach is pulled through the gastrocolic ligament, particularly in cases of transverse colostomy, as observed in the present case. The other is *laxity type*, related to the laxity of the peritoneal ligaments that normally tether the stomach to adjacent organs, such as the gastrocolic, gastrosplenic, gastrophrenic, and hepatogastric ligaments, which may occur with any left-sided stoma. This mechanism represents a more common type of parastomal hernia in which the parastomal opening allows protrusion of intra-abdominal organs with greater mobility, such as the small intestine or omentum.

In the surgical management of gastric incarceration of *traction type* associated with a transverse colostomy, several technical considerations should be addressed. To ensure that the parastomal defect is completely covered with a mesh, the stomach must be kept at a safe distance from the stoma. Furthermore, to prevent recurrence, it is important to avoid traction on the stomach by the colon and mesocolon. Therefore, we performed a meticulous dissection adjacent to the hernial orifice, entered the lesser sac, divided the left gastrocolic ligament, and further dissected the stomach from the transverse mesocolon.

## CONCLUSIONS

We reported a rare case of gastric incarceration in a parastomal hernia of a transverse colostomy, which was successfully managed with laparoscopic repair using the Sugarbaker technique. Intraoperatively, adequate stomach mobilization, including dissection from the transverse mesocolon and division of the gastrocolic ligament, was a key step in ensuring mesh coverage of the hernia defect and minimizing recurrence risk.

## DECLARATIONS

### Availability of data and materials

The datasets supporting the conclusions of this article are included within the article.
